# Comparison of tumor microenvironment in primary and paired metastatic ER+/HER2- breast cancers: results of a pilot study

**DOI:** 10.1186/s12885-021-07960-z

**Published:** 2021-03-10

**Authors:** Annalisa Zeppellini, Stefania Galimberti, Biagio Eugenio Leone, Claudia Pacifico, Francesca Riva, Federica Cicchiello, Serena Capici, Claudia Maggioni, Luca Sala, Marina Elena Cazzaniga

**Affiliations:** 1 Department of Medical Oncology, ASST Monza, via Pergolesi, Monza, Italy; 2 School of Medicine and Surgery, Bicocca Bioinformatics Biostatistics and Bioimaging B4 Center, University of Milano – Bicocca, via Cadore, Monza, Italy; 3 School of Medicine and Surgery, University of Milano – Bicocca, via Cadore, Monza, Italy; 4 Department of Medical Pathology, ASST Monza, via Pergolesi, Monza, Italy; 5 Phase 1 Research Centre - ASST Monza, via Pergolesi, Monza, Italy

**Keywords:** Breast Cancer, Luminal, Microenvironment, Tumor infiltrating lymphocytes (TILs), T CD8 +, T CD4+ FOXP3+

## Abstract

**Background:**

Tumor microenvironment (TME) is a dynamic setting and changes in TILs and their subpopulations are potential candidates to influence the metastatic process. Aim of this pilot study is to describe the changes occurring between primary breast cancers and their paired metastases in terms of TILs composition. To assess if these changes influence the process of metastasis development, we used a control group of patients.

**Methods:**

We retrospectively identified 18 Luminal patients, for whom primary and metastatic tissue were available (cases) and 18 paired-matched patients (controls), not relapsed after at least 9 years of follow-up, and we quantified TILs and their composition (i.e. T CD8+ and CD4+/FOXP3+). The presence of TILs was defined as ≥10%.

**Results:**

Our results showed that the microenvironment composition of relapsed patients was poor of TILs (median = 5%, I-III quartiles = 0.6–5%), CD8+ (2.5%, 0–5%) and CD4+/FOXP3 + (0%, 0–0.6%) in the primary tumor. Comparable results were observed in their related metastases (TILs 3.8%, 0.6–5%; CD8+ 0%, 0–1.3%; CD4+/FOXP3+ 0%,0–1.9%). On the contrary, the microenvironment in the control group was richer of TILs (5%, 5–17.5%) in comparison to cases, both in primary tumor (*p* = 0.035) and related metastases (*p* = 0.018). Although CD8+ in controls were similar to cases at primary tumor (*p* = 0.6498), but not at metastasis (*p* = 0.0223), they expressed only one part on the TILs subpopulations (*p* = 0.0060), while TILs in the cases at primary tumor were almost completely CD8+ (*p* = 0.5034).

**Conclusions:**

These findings suggest that the lack of activation of immune system in the primary tumor might influence the multifactor process of cancer progression.

## Background

The tumor microenvironment (TME) is composed by a heterogeneous group of elements present in the stroma: fibroblasts, cells of the immune system, extracellular matrix, cytokines and macrophages. These cells play a central role in shaping the microenvironment, driving anti- or pro-tumor activity. All these elements can potentially interact with cancer cells and their interaction be part of the carcinogenesis process [1]. Tumors engage the immune system from the beginning: initially involving the cells of the innate system, such as macrophages and mast cells, and subsequently cells of the acquired system, particularly T cells [2]. Recent studies have confirmed the important role of tumor infiltrating lymphocytes (TILs) in tumor progression, which are mostly composed by T CD4+ and T CD8+ [3]. Lymphocytes interact with tumor cells, in a similar way as they interact with antigen presenting cells (APC): T CD4+, activated in T Helper, secrete cytokines that induce proliferation and stimulation of macrophages, B and T lymphocytes and help CD8+ lymphocytes activation in Cytotoxic T Lymphocytes (CTL). These latter, when activated, promote direct mechanisms of tumor cells lysis.

In breast cancer (BC), extensive tumor infiltration by cytotoxic CD8+ T cells is strongly associated with patient survival and response to therapy [4]. In contrast, the pro- or anti-tumor activity by T CD4+ depends on their subpopulations: Th1 cells (the principal cellular source of interferon-γ) have been related with favourable clinical outcomes and Tfh (T follicular helper cells, the newest CD4+ subset) was positively associated with patient outcome both in the adjuvant and neoadjuvant settings [5]. Differently, the Th2 cells have been reported to be associated with dampening of the antitumor response [6] and the presence of CD4+ regulatory T cells (Tregs, which immunohistochemistry (IHC) marker is FOXP3) has been associated with worse prognosis due to their negative immunoregulatory activity [7]. TILs are generally associated with favourable prognosis in BC [8]. These findings are consistent in prognostic and predictive terms in triple negative and HER2 positive tumors [9–12], whereas few and sometimes controversial results are available regarding the role of TILs in luminal BC (L-BC), especially in advanced disease. Montagna et al. showed that high TILs levels are significantly associated with a worse Time to Progression (TTP) in L-BC treated with metronomic chemotherapy [13] and Denkert showed that elevated TILs are related with a worse survival [14]. In contrast, Jang suggested that TILs had a favourable prognostic role in luminal B BC [15]. Limitations in all these studies were methods of TILs determination, different cut-off values to define the presence of TILs and the unavailability of data on TILs subpopulations.

Aim of this pilot study is to describe TME changes between primary tumors and their paired metastases in terms of TILs composition in L-BC. To assess if these changes influence the process of metastasis development, we used a control group of patients, matched by stage and luminal status without evidence of relapse during long term follow-up. In addition, TILs subpopulations were analysed as a spectrum of positive and negative immunomodulatory activity inside tumor.

## Methods

### Patients

We retrospectively retrieved from the archive of the Pathology Division (San Gerardo Hospital, ASST Monza, Italy) specimens of patients affected by invasive L-BC and treated at the Department of Medical Oncology from February 2002 to January 2018. Since the samples were collected in different years, during which revisions have been made to redefine the luminal status, we used an univocal classification, referring to the definition of the St Gallen International Expert Consensus of 2013 [16].

Cases were defined as patients with Luminal (ER+/PgR+ or ER+/PgR-) metachronous metastases with pathological reports of both the primary tumor and the metastasis. Additional inclusion criteria were persistence of Luminal A/B characteristics in the biopsy of metastasis (i.e. biopsy done before starting any therapy for advanced BC to avoid any influence on the tumor microenvironment). Patients with synchronous metastasis or discordant receptor status were thus excluded. In order to assess the impact of TME on relapse, we identified as controls Luminal patients without evidence of tumor relapse for at least 9 years from diagnosis, who were individually matched with cases according to the anatomopathological characteristics of the primary tumor, i.e. luminal status (A/B), tumor size and lymph node status (T and N). The interval of follow-up was established looking at the time of relapse of L-BC [17] and considering that the risk of relapse becomes very low after 8 years from diagnosis. So far, we identified 18 patients with diagnosis of L-BC that became metastatic and 18 matched women without long term evidence of tumor relapse. Follow-up was updated as of February 2019.

The study was approved by the local Ethical Committee and performed in accordance with the ethical standards as laid down in the 1964 Declaration of Helsinki and its later amendments or comparable ethical standards. An informed consent was obtained from all participants.

### Pathological assessment

**Fig. 1:**
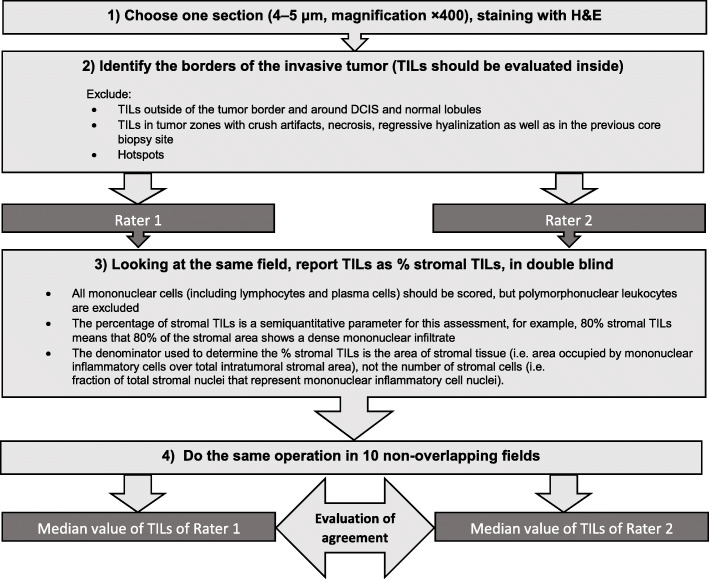
Flow-chart of the method of TILs determination

**Fig. 2:**
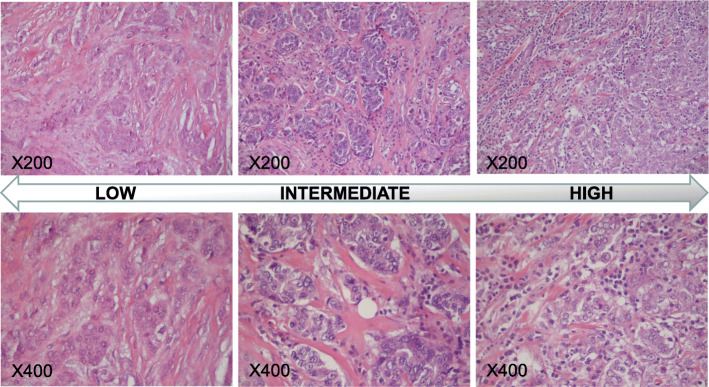
TILs in H&E staining. Examples of TILs in the three-categories we used: Low (< 10%), Intermediate (10–50%) and High (≥ 50%). Magnification × 200, × 400

**Fig. 3:**
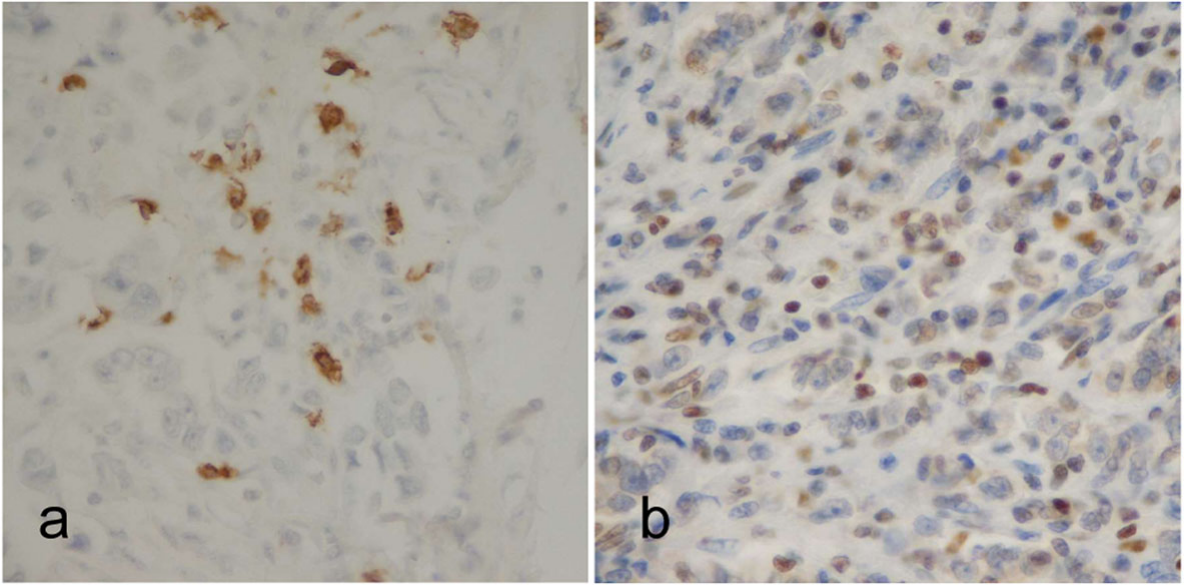
Representative immunohistochemical expression of **a**) T CD8+ and **b**) T CD4+ FOXP3+. Magnification × 400

We derived 4-μm-thick sections from paraffin blocks for Haematoxylin and Eosin (H&E) and IHC stainings. The samples were independently assessed by two different raters (A.Z. and B.E.L.) blinded to patients’ stage. Ten non overlapping fields (magnification × 400) were selected according with the rules defined by the International TILs Working Group [18]. TILs were expressed as percentage of immune cells in each field and summarised in the median value. A flow-chart summarising the approach used for TILs determination is shown in Fig. 1. A three-categories classification was used: Low (< 10%), Intermediate (10–50%) and High (≥ 50%), with a value ≥ 10 indicating the presence of TILs (Fig. 2). These limits were defined according to the cut-off values reported in the most recent literature [9, 10, 13, 19–22]. The same method (i.e. measurements in the same 10 fields for each rater in double blind) was used for CD8 and CD4 FOXP3 stainings. A representative image of immunohistochemical expression of T CD8+ (monoclonal antibody C8/144B, Dako) and T CD4+ FOXP3+ (monoclonal antibody 5H10L18, Invitrogen) is shown in Fig. 3.

### Statistical analyses

Mean and standard deviation or quartiles were used for descriptive purposes, as appropriate. Characteristics at diagnosis in the two groups of L-BC patients were compared by means of a chi-square test for categorical variables and a t-test for continuous ones. The inter-observer agreement in measuring TILs in continuous was analysed according to the Bland-Altman method and the 95% limits of agreement were calculated. The weighted Cohen’s kappa index was used to assess the concordance between operators to classify TILs into the 3 ordered categories. Non parametric tests were used to compare TILs (and their subpopulations) in paired (Wilcoxon signed-rank test) or independent samples (Mann-Whitney test). All tests were 2-sided and with a significance level α = 0.05.

## Results

### Patients characteristics

The characteristics at diagnosis of the primary breast tumor in the 18 metastatic L-BC patients and their matched non metastatic L-BC controls are summarized in Table 1*.* The 36 L-BC patients were mainly T1 (50%), N+ (66.6%), stage II-III (61.1%) and luminal A (63.9%). Adjuvant chemotherapy (CT) was received by 66.7% and 77.8% of the women in the metastatic and in the control group, respectively, and almost all patients have done adjuvant endocrine therapy (HT) (94.4% vs 100%). The mean age at diagnosis in the two groups was similar: 64.5 years (sd = 16.7) in the metastatic patients vs 64.3 years (sd = 14.1) in the control group.

**Table 1:** Clinical characteristics at diagnosis of primary BC in the 18 patients who subsequently became metastatic (Cases) and in the 18 matched non metastatic patients (Controls). The *p*-values for comparison were not reported for the matching factors

Patients characteristic	Cases		Controls		Overall		***p***
	(***n*** = 18)		(***n*** = 18)		(***n*** = 36)		
	n	(%)	n	(%)	n	(%)	
**T (primary)**							
**1**	9	(50.0)	9	(50.0)	18	(50.0)	
**2**	8	(44.4)	8	(44.4)	16	(44.4)	
**3**	1	(5.6)	1	(5.6)	2	(5.6)	
**N (positive lymph-nodes)**							
**0**	6	(33.3)	6	(33.3)	12	(33.3)	
**1**	5	(27.8)	5	(27.8)	10	(27.8)	
**2**	4	(22.2)	4	(22.2)	8	(22.2)	
**3**	3	(16.7)	3	(16.7)	6	(16.7)	
**Stage at diagnosis**							
**I**	4	(22.2)	4	(22.2)	8	(22.2)	
**II**	7	(38.9)	7	(38.9)	14	(38.9)	
**III**	7	(38.9)	7	(38.9)	14	(38.9)	
**Luminal**							
**A**	12	(66.7)	12	(66.7)	24	(66.7)	
**B**	6	(33.3)	6	(33.3)	12	(33.3)	
**Grading**							0.5361
**1**	2	(11.1)	2	(11.1)	4	(11.1)	
**2**	9	(50.0)	12	(66.7)	21	(58.3)	
**3**	7	(38.9)	4	(22.2)	11	(30.6)	
**Adjuvant CT**							0.4088
**No**	6	(33.3)	4	(22.2)	10	(27.8)	
**Antracycline**	5	(27.8)	3	(16.7)	8	(22.2)	
**Antracycline + taxanes**	7	(38.9)	11	(61.1)	18	(50.0)	
**Adjuvant HT**							0.2146
**No**	1	(5.9)	0	(0)	1	(2.9)	
**Tamoxifen ± LHRH an**	8	(47.1)	3	(16.7)	11	(31.4)	
**Letrozol**	5	(29.4)	10	(55.5)	15	(42.9)	
**Anastrozol**	3	(17.6)	4	(22.2)	7	(0.2)	
**Sequence of HT**	0	(0)	1	(5.6)	1	(0.03)	
	**mean**	**sd**	**mean**	**sd**	**mean**	**sd**	
**Age (years)**	64.5	16.7	64.3	14.1	64.4	11.7	0.9554

*CT* chemotherapy, *HT* hormone-therapy, *sd* standard deviation

The characteristics of metastatic lesions and treatments performed by the 18 patients with a recurrent breast cancer after a median of 6.1 years (I-III quartiles = 4.0–8.9 years) from the first diagnosis are summarized in Table 2. Sites of biopsied recurrences were distant metastases in 12 cases (66.7%) and locoregional soft tissue or lymph nodes in 6 cases (33.3%). All metastatic patients did a systemic therapy (HT/CT) for advanced disease.

**Table 2:** Site of biopsy of metastatic lesions and number and type of treatments for advance setting

Patients characteristics	Metastatic cases (***n*** = 18)	
	n	(%)
**Site of biopsy**		
**lymph node**	4	(22.2)
**liver**	4	(22.2)
**bone**	3	(16.6)
**skin**	1	(5.6)
**pleura**	2	(11.1)
**ovary**	2	(11.1)
**lung**	1	(5.6)
**breast**	1	(5.6)
**Max/last line of palliative CT**		
**No CT**	11	(61.1)
**III line**	2	(11.1)
**≥ IV line**	5	(27.8)
**Max/last line of palliative HT**		
**No HT**	7	(38.9)
**I line**	6 (2^a^)	(33.3)
**II line**	3 (1^a^)	(16.7)
**III line**	2 (1^a^)	(11.1)
**Max/last Line of treatment for advanced disease**		
**I**	6 (3^a^)	(33.3)
**II**	3 (1^a^)	(16.7)
**III**	3 (2^a^)	(16.7)
**IV-IX**	6 (2^a^)	(33.3)

^a^ongoing

With an overall median follow-up of 9.7 years, 29 out of the 36 patients in the study were alive: eleven women were still receiving active therapies for metastatic BC, while all the 18 patients in the control group were relapse-free. Only one patient in this latter group was still in treatment with aromatase inhibitor at that end of follow-up, while the remaining had stopped hormone therapy after the standard 5 years of treatment.

### TILs

**Fig. 4:**
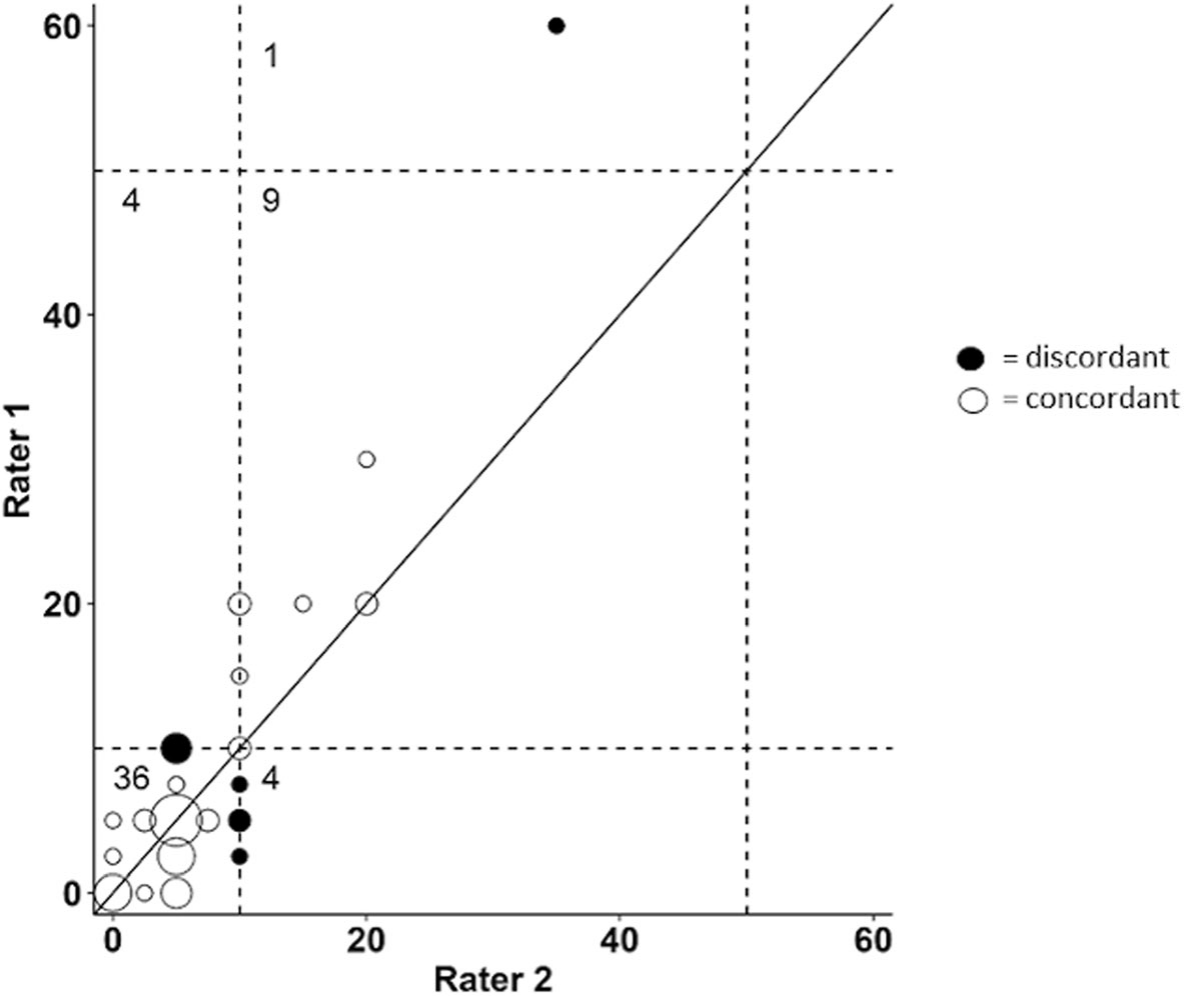
Agreement of two independent raters in quantifying TILs of 54 Luminal-BC specimens. The solid line is the line of equality, while the dashed lines represent the threshold for the three groups TILs classification: Low (< 10%), Intermediate (10–50%) and High (≥50%). The numbers indicate the absolute frequency of specimens classified by the two raters and the size of the circles is proportional to the frequency of specimens sharing the same TILs values

The agreement between raters, evaluated through the Bland-Altman analysis performed on TILs as a continuous variable, resulted in a low bias: - 0.7%, (95% limits of agreement = - 10.7%; 9.3%). When the concordance was assessed using the classification in three categories, we had 45 correctly classified patients out of 54 (83.3%), with a weighted Cohen k-index of 0.59 (95% CI = 0.36–0.81). Discrepancies were mostly borderline misclassifications in the low-intermediate categories due to the cut-off value of 10%, with only one marked error in the high-intermediate classes (Fig. 4).

**Fig. 5:**
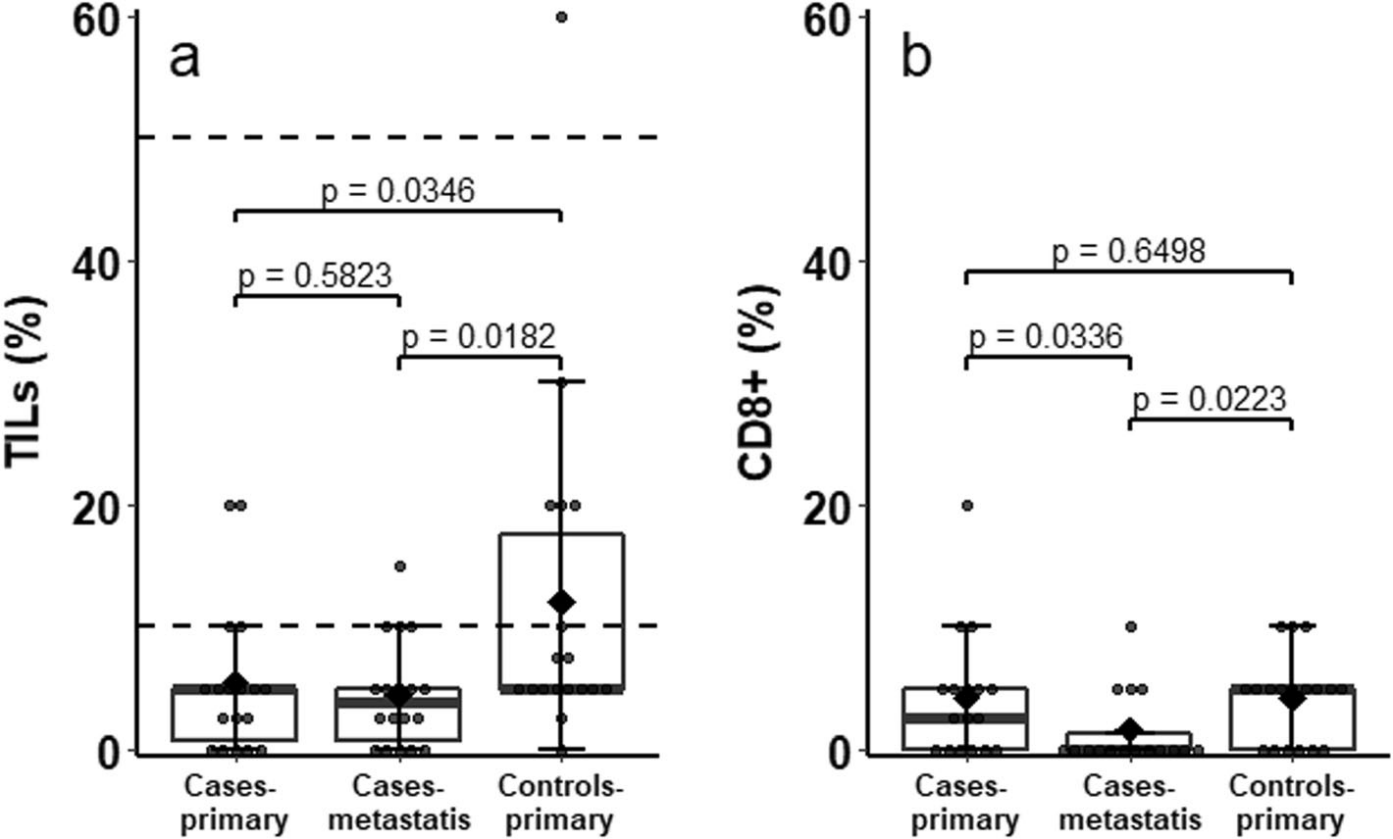
Box and whisker plot by groups of **a**) TILs (the dashed lines represent the threshold for the three group TILs classification: Low (< 10%), Intermediate (10–50%) and High (≥50%)), and **b**) T CD8+. The box contains data that fall between the first and third quartiles, the horizontal line indicates the median, the diamond indicates the mean, and the brackets delineate 1.5 times the interquartile range. Individual data are also shown, with points outside the brackets classified as outliers. Comparisons within primary tumor and metastasis of relapsed patients are obtained from the Wilcoxon signed-rank test, while those between primary tumor of relapsed patients and controls are obtained from the Mann-Whitney test

The median value of TILs measured by rater 1 in the primary tumor was 5% both in women who subsequently became metastatic and in the matched control group of non-metastatic patients. However, the two distributions were significantly different (*p* = 0.0346), with I-III quartiles of 0.6–5% (range = 0–20%) and 5–17.5% (range = 0–60%), respectively (Fig. 5a and Table 3). No changes in the microenvironment inside the same patient were observed from primary tumor to related metastasis (median = 3.8%, I–III quartiles = 0.6–5%, *p* = 0.5823). The comparison between the TILs values in primary tumor of controls and metastases of relapsed patients was statistically significant (*p* = 0.0182). TILs were positive (i.e. ≥10%) in the 22.2% of cases (both in primary tumor and metastasis) and in the 33.3% of non-metastatic L-BC controls (Fig. 5a and Table 4). Similar findings were observed for rater 2, as shown in Table 3*.*

**Table 3:** Descriptive analysis of TILs, T CD8+ and T CD4+/FOXP3+ evaluated in 18 metastatic patients (both at primary tumor and at metastasis) and their 18 matched controls by two independent raters

Rater	Characteristic		Group		
			Cases-primary tumor	Cases-metastasis	Controls-primary tumor
**1**	**TILs (%)**	**Min-Max**	0–20	0–15	0–60
		**Median**	5	3.8	5
		**I-III Q**	0.6–5	0.6–5	5–17.5
	**CD8**+ **(%)**	**Min-Max**	0–20	0–10	0–10
		**Median**	2.5	0	5
		**I-III Q**	0–5	0–1.3	0–5
	**FOXP3**+ **(%)**	**Min-Max**	0–5	0–5	0–10
		**Median**	0	0	0
		**I-III Q**	0–0.6	0–1.9	0–5
**2**	**TILs (%)**	**Min-Max**	0–20	0–10	0–35
		**Median**	5	5	7.5
		**I-III Q**	5–5	1.3–5	5–10
	**CD8**+ **(%)**	**Min-Max**	0–10	0–10	0–15
		**Median**	0	0	5
		**I-III Q**	0–5	0–0.6	2.5–6.9
	**FOXP3**+ **(%)**	**Min-Max**	0–10	0–10	0–10
		**Median**	0	1.3	0
		**I-III Q**	0–3.1	0–5	0–3.8

*Q* quartiles

**Table 4:** Distribution of TILs categories in cases and controls: Low (< 10%), Intermediate (10–50%) and High TILs (≥50%)

TILs	Cases-primary tumor (***n*** = 18)		Cases-metastasis (***n*** = 18)		Controls-primary tumor (***n*** = 18)	
**Rater 1**	**n**	**(%)**	**n**	**(%)**	**n**	**(%)**
**Low**	14	(77.8)	14	(77.8)	12	(66.7)
**Intermediate**	4	(22.2)	4	(22.2)	5	(27.7)
**High**	0	(0)	0	(0)	1	(5.6)
**Rater 2**	**n**	**(%)**	**n**	**(%)**	**n**	**(%)**
**Low**	14	(77.8)	16	(88.9)	10	(55.6)
**Intermediate**	4	(22.2)	2	(11.1)	8	(44.4)
**High**	0	(0)	0	(0)	0	(0)

### TILs subpopulations (T CD8+ and T CD4+ /FOXP3+)

The distribution of positive immunomodulatory activity of CD8+ (Table 3, Fig. 5b) in the control group (median = 5% and I-III quartiles = 0–5%) is similar to that of the primary tumor of cases (median = 2.5% and I-III quartiles = 0–5%, *p* = 0.6498), while a statistically significant decrease was observed with regard to the activity inside metastasis that was almost absent (median = 0% and I-III quartiles = 0–1.3%, comparison of primary tumor vs metastasis *p* = 0.0336 and controls vs metastasis of cases *p* = 0.0223). In addition, since CD8+ are a TILs subpopulation, we compared their distributions within cases at primary tumor and controls (Fig. 5a and b). A significant difference was found for controls (*p* = 0.0060), while the TILs and CD8+ distributions are completely overlapping in cases (*p* = 0.5034).

The median value of cells FOXP3+ was 0% in all groups with slight differences in the distribution of data (primary tumor of cases: I-III quartiles = 0–0.6%; primary tumor of controls: I-III quartiles = 0–5%; metastasis: I-III quartiles = 0–1.9%). The results on these parameters are consistently seen by the second rater (Table 3).

## Discussion

The role of TILs in luminal breast cancers (L-BC) has not been clearly defined yet, especially in advanced setting. According with the concept of immunoediting [23], we studied whether there were differences in TME between primary and their paired metastatic localizations.

We observed that TILs were almost absent in the L-BC that develop metastases both at primary tumor (median = 5%) and at metastasis (median = 3.8%; *p* = 0.5823) and that the microenvironment of these patients was exactly the same in primary and metastatic samples, with 77.8% of Low (< 10%), 22.2% Intermediate (10–50%) and 0% High (≥50%) TILs levels. Our findings are similar to those reported in the literature: TILs are generally low in L-BC, due to their lower immunogenicity, derived also by lower chromosomal instability, that is a stimulus to a more conspicuous release of tumor antigens, which would result in better activation of the immune system [24]. Similar findings were reported by Sobottka et al., suggesting that the TME of primary tumor reflected the metastatic site [25]. A slight lower percentage of TILs was seen in primary tumor as compared to metastasis by Cimino-Mathews et al. [26], suggesting that immune escape might play a role in tumor progression. Differently, Zhu et al. [27], by analysing RNA sequencing data from 50 primary breast tumors and their patient-matched metastatic tumors (METs), reported that METs had a significantly lower abundance of total immune cells, including CD8+ T cells, regulatory T cells and dendritic cells. An exception was M2-like macrophages, which were significantly higher in METs across the organ sites examined, but further studies are needed to explain their role. However, tumour samples were not selected according to hormone receptor (HR) status, whereas we analysed patients with only L-BC.

TILs are anyway present in L-BC, especially in Luminal B, although with a lower expression if compared to non-Luminal cancers [19, 26]. To corroborate this aspect and assess the potential role of TILs in the metastatic process, we compared the microenvironment of the primary tumor of metastatic patients with that of a control group of non-metastatic patients, matched for biological and clinical characteristics of the primary tumor (i.e WHO stage and luminal A/B status). Although the median of TILs values was of 5% both in the primary tumor of cases and controls, the two distributions were quite different in terms of variability (Fig. 5a) and their comparison resulted statistically significant (*p* = 0.0346).

High values of T CD8+ are associated with a better prognosis in BC, regardless of histologic subtypes [4]. In our study, T CD8+ cells were almost completely absent in the microenvironment of the metastases (median = 0%) and very low, although significantly different (*p* = 0.0336), in the corresponding primary tumors (median = 2.5%) suggesting a mild reduction of the anti-tumoral activity of immune system in the metastatic process. These results are comparable with those in the literature that showed that CD8+ and CD4+ cells significantly decreased from primary to metastatic tumors [7]. Savas et al. [28] performed single-cell RNA sequencing (scRNA-seq) of 6311 T cells isolated from human BCs and showed that significant heterogeneity exists in the infiltrating T cell population. They demonstrated that BCs with a high number of TILs contained CD8+ T cells with features of tissue-resident memory T (TRM) cell differentiation and that these CD8+ TRM cells expressed high levels of immune checkpoint molecules and effector proteins. A CD8+ TRM gene signature developed from the scRNA-seq data was significantly associated with improved patient survival in early-stage triple-negative breast cancer (TNBC) and provided better prognostication than CD8 expression alone. A recent study [29] also demonstrated that Luminal B shows a high antigenicity and T cell clonality, yet a low abundance of CD8 TILs. In contrast, Luminal A shows a low antigenicity and a poorness of CD8 TILs, which associates with T cell influx parameters, such as expression of adhesion molecules. Authors concluded that it is not only the presence of CD8 T cells, but rather T cell clonality, T cell subset distribution, co-inhibition and antigen presentation that reflect occurrence of a CD8 T cell response in BC subtypes, providing a rational for subtype-specific combination immune therapies. In our study, cases and controls had similar CD8+ in the primary tumor (median = 2.5 vs 5%, *p* = 0.6498), but what was very suggestive is that TILs were completely absorbed by CD8+ in the cases (*p* = 0.5034), but not in the controls (*p* = 0.0060), where there was room for other elements of the immune system, and this might explain their long time without progression. However, this is only a speculation that needs further mechanistic studies. Finally, the median value of cells FOXP3+ was 0% in both primary tumor and metastases, and these results are similar to the findings already reported, which generally describe the absence of FOXP3+ in L-BC [7].

In this study we performed a double-blind quantification of TILs and showed a high inter-observer reproducibility, with a very limited mean bias of - 0.7% (95% limits of agreement = - 10.7%; 9.3%). The determination of the quantitative agreement between two different operators is one of the novelties of our study, given that results are generally reported only in terms of the interclass correlation coefficient in the literature. A recent study on BC is in line with our finding on the Cohen index in L-BC, remarking that the IWTILG recommendations were reproducible and reliable [30]. The conclusion that the visual evaluation of TILs is operator independent is a very important point that strengthen our findings, despite the literature is going towards computationally heavy image analysis to provide more standardized and efficient TILs quantification with a costly price to pay [31].

## Conclusions

In conclusion, the results of our study are very promising and suggestive of a possible active role of the immune system in the relapsing process, but we recognize that the main limitation of our pilot study was the small sample size that is due to the rarity of the biopsies done before the treatment for advance setting. This aspect is of paramount importance since all the samples of metastatic sites were naïve from CT/HT for advanced disease, allowing to preserve data of microenvironment from alterations due to therapies. There are similar studies assessing the changes of tumor microenvironment from primary tumor to related metastasis in advanced breast cancers. However, at our knowledge, this is the first study focused on Luminal metastatic breast cancer considering a control group of L-BC who didn’t show any evidence of relapse after 9 years of follow-up. Further and more extended evaluations are needed to understand the prognostic role of TILs in L-BC, but this study is an initial observation of the potential active role of immune system also in this type of cancer.

## Data Availability

The datasets used and/or analysed during the current study are available from the corresponding author on reasonable request.

## Abbreviations

TILs: Tumor infiltrating lymphocytes
BC: Breast cancer
TME: Tumor microenvironment
APC: Antigen Presenting Cells
T CD4+ FOXP3+: Lymphocytes T CD4+ FOXP3+
T CD8+: Lymphocytes T CD8+
CTL: Cytotoxic T Lymphocytes
Tregs: Regulatory T cells
IHC: Immunohistochemistry
L-BC: Luminal breast cancer
TTP: Time to Progression
H&E: Haematoxylin and Eosin
CT: Chemotherapy
HT: Hormone Therapy
Mets: Metastasis
HR: Hormone Receptor
TRM: Tissue-resident memory
TNBC: Triple Negative Breast Cancer
